# Dynamic insights into research trends and trajectories in early reading: an analytical exploration via dynamic topic modeling

**DOI:** 10.3389/fpsyg.2024.1326494

**Published:** 2024-02-07

**Authors:** Ting Wang, Hanqing Xu, Chenyuan Li, Fan Zhang, Jiaoping Wang

**Affiliations:** ^1^College of Science and Technology, Ningbo University, Cixi, China; ^2^Ningbo Childhood Education College, Ningbo, China

**Keywords:** early reading, dynamic topic model, topic identification, topic evolution analysis, visualization

## Abstract

**Introduction:**

Early reading has gained significant attention in the academic community. With the increasing volume of literature on this subject, it has become crucial to assess the current research landscape and identify emerging trends.

**Methods:**

This study utilized the dynamic topic model to analyze a corpus of 1,638 articles obtained from the Web of Science Core Collection to furnish a lucid understanding of the prevailing research and forecast possible future directions.

**Results:**

Our in-depth assessment discerned 11 cardinal topics, among which notable ones were interventions' impacts on early reading competencies; foundational elements of early reading: phonological awareness, letters, and, spelling; and early literacy proficiencies in children with autism spectrum disorder. Although most topics have received consistent research attention, there has been a marked increase in some topics' popularity, such as foundational elements of early reading and early literary proficiencies in children with autism spectrum disorder. Conversely, other topics exhibited a downturn.

**Discussion:**

This analytical endeavor has yielded indispensable insights for scholars, decision-makers, and field practitioners, steering them toward pivotal research interrogatives, focal interest zones, and prospective research avenues. As per our extensive survey, this paper is a pioneering holistic purview of the seminal areas of early reading that highlights expected scholarly directions.

## 1 Introduction

Early reading has received global recognition due to its paramount importance and profound connection with young children's academic trajectories and subsequent life outcomes (García and Weiss, 2017). A robust body of research has consistently indicated that early proficiency in reading skills predisposes children to academic excellence (Young and Dolzhenko, 2022; Muroga et al., 2023). In contrast, those encountering early reading challenges often experience Matthew effects, where initial reading difficulties lead to increasingly pronounced disparities in both reading proficiency and broader academic accomplishments (Stanovich, 2009).

Despite the global consensus on the significance of early reading, a comprehensive review that encapsulates the knowledge structure and thematic evolution of early reading at an international level is noticeably lacking. This lack not only hinders a holistic understanding of the field but also limits the potential for identifying emergent trends and critical topics that could guide future research and practice. Addressing this gap is not merely an academic endeavor but a crucial step in influencing the trajectory of early reading research and its practical applications. Therefore, the present study was driven by the following fundamental research question: Which key topics and trends define the knowledge structure and topic evolution in early reading?

To explore this question, we employed the dynamic topic model (DTM) to critically assess relevant scientific literature. This method allowed us to delve into prevalent knowledge topics and their evolutionary patterns within the early reading field. Compared to traditional systematic literature reviews, the DTM, which is grounded in text mining techniques, enables a more comprehensive and dynamic analysis of domain-specific knowledge (Xiong et al., 2023). It is particularly adept at capturing and analyzing long-term trends and gradual shifts in knowledge structures, thus providing deeper insights into the historical development, current state, and potential future directions of early reading.

Through this inquiry, we aimed to furnish the early reading discipline with invaluable insights and offer a discerning perspective on its prospective future directions. This study sought to not only fill a critical gap in the literature but also serve as a foundational resource for scholars, educators, and policymakers dedicated to enhancing early reading outcomes across diverse contexts.

## 2 Literature review

Early reading refers to the process in which young children develop foundational language skills during the preschool and early primary school years through word recognition and comprehension of written text. It plays a crucial role in language acquisition and in young children's overall development (Niklas et al., 2016). This stage encompasses all experiences and encounters young children have with oral and written conversations, stories, books, and printed materials. It is essential for their development of a rich vocabulary, self-expression, and reading comprehension abilities (Barron, 1986; Lennox, 2013). Early reading not only lays the foundation for young children's school readiness and lifelong learning but also profoundly affects their cognitive, emotional, and social development. It establishes the groundwork for acquiring knowledge in other subject areas (Vanbecelaere et al., 2023) and significantly influences future academic achievements and educational attainment (Cunningham and Stanovich, 1997; Duncan et al., 2007). Therefore, gaining a comprehensive understanding of early reading's multidimensional construction and its pivotal role in language learning and child development holds paramount significance in designing effective educational strategies and intervention measures.

Precisely due to early reading's pivotal role in language acquisition and child development, the academic community has paid close attention to this field and has conducted extensive research on the various internal and external factors that influence early reading development. Researchers have specifically examined the impact of cognitive abilities (Hernández Finch et al., 2014), language skills (Clayton et al., 2020), reading motivation and interest (Altun, 2019), and reading strategies and techniques (Bojczyk et al., 2016) on early reading skills development. These studies have revealed internal factors' significant influence on the formation of early reading abilities and have provided profound insights into the fundamental elements of early reading development. Concerning external environmental factors, scholars have focused on the impact of the school environment (Taylor et al., 2010), the home environment (Hamilton et al., 2016), gene–environment interaction (Olson et al., 2014), and reading materials (Luo et al., 2020) on early reading abilities. These studies have emphasized the external environment's crucial role in nurturing early reading development and have highlighted the school and family environments' supportive role in facilitating early reading progress.

In recent years, some scholars have conducted review studies of the early reading field that describe its current state and development characteristics. For instance, Allington and McGill-Franzen (2021) reviewed the relationship between reading volume and reading achievement. Ostiz-Blanco et al. (2021) systematically reviewed the use of electronic interventions to improve early first-language reading. Arciuli and Bailey (2021) systematically organized the factors affecting early reading development in children with autism spectrum disorder (ASD). Lorio et al. (2022) systematically reviewed intervention strategies for infants and toddlers who engage in reading with their parents. Hall et al. (2023) systematically reviewed the impact of kindergarten writing instruction on literacy skills. Although these review papers have provided valuable insights into the development of knowledge topics in the field of early reading, they also have certain limitations. For example, they have tended to focus on specific areas (e.g., the type of book media) or specific groups of children (e.g., children with ASD). Additionally, they have relied on qualitative meta-analyses or systematic review methods and used small sample sizes; the research results therefore lack persuasive power. Furthermore, the use of manual coding methods for literature screening, classification, and analysis means that the research results depended on the researchers' subjectivity, which may have resulted in untrustworthy research outcomes. Therefore, to assess early reading development trends more accurately, use of advanced quantitative research methods and more comprehensive data sources is needed.

With the rapid progression of artificial intelligence technologies, traditional analysis methods based on external features (e.g., keyword and citation networks) are now ill-equipped to handle high-dimensional data and address intricate challenges. Consequently, researchers have begun to adopt newer natural language processing (NLP) methodologies to investigate scientific documents in a more nuanced manner. These methods are better suited to address the challenges of high-dimensional data and intricate issues as they facilitate profound analyses and understanding of scientific documents and thereby offer comprehensive support to scientific inquiry. For instance, Chen et al. (2020) employed the structural topic model to identify topics in 3,342 articles published in *Computers & Education*, a leading educational technology journal, with the aim of identifying major research topics and potential future directions in educational technology. Kaushik et al. (2023) utilized the latent Dirichlet allocation (LDA) model to mine topics from 3,844 articles related to social entrepreneurship that were obtained from the Scopus and Web of Science (WOS) databases and identify latent research topics and future development trends in social entrepreneurship studies. Wang et al. (2023) also used the LDA to mine topics from 8,197 articles related to health-promoting behaviors among miners that were obtained from the WOS database to assist researchers with grasping the core research topics and potential future directions in that domain. Evidently, NLP techniques have been embraced across various research domains, and the feasibility of using topic modeling to extract and observe the distribution of topics and research trends in extensive literature has been empirically validated.

Hence, this study employed an NLP-based dynamic topic modeling approach to extract key information from paper titles, keywords, and abstracts and autonomously generate research knowledge topics within the dataset and facilitate a comprehensive analysis of topic distribution characteristics and evolving trends in early reading. The research outcomes are expected to aid researchers, educators, and policymakers in gaining a deeper understanding of hotspots, trends, and potential future directions in early reading. This paper provides profound insights and holistic support for scientific investigations in the early reading domain and further propels the advancement of early reading education through the refinement of educational content and the shaping of related policies.

## 3 Methods and materials

### 3.1 Research approach

This study utilized journal articles obtained from the WOS Core Collection as a data source and employed the DTM to dissect the characteristics of the knowledge structure and the topic evolution process in early reading research data from two dimensions. The detailed research steps were as follows. Firstly, literature retrieval entailed retrieving scholarly early childhood reading literature from the WOS database. Secondly, data preparation entailed extracting titles, keywords, and abstracts from the literature to serve as the corpus data, then cleaning the data and segmenting them into chronological time windows. Third, topic analysis entailed determining the number of topics by assessing topic consistency and conducting a horizontal analysis of the initial results of the DTM. Fourth, the evolution of trends was ascertained by calculating the topic heat for the various time windows, visualizing topic evolution trends, and analyzing the evolution path of topic keywords at a granular level.

### 3.2 DTM

Traditional largescale literature analysis methods often employ co-citation and keyword networks for content mining and analysis. However, co-citation network analysis fails to capture the latest research content as co-citation relationship formation requires time. On the other hand, keyword network analysis faces the issue that many articles do not include keywords, or the keywords are selected from a predefined list. Hence, the displayed keywords may not always accurately reflect the articles' content.

To overcome these issues, we opted for the topic modeling approach. Compared to co-citation and keyword analysis, topic modeling has demonstrated greater adaptability and efficiency in providing comprehensive content analysis (Kuhn, 2018). The topic modeling method involves inferring and modeling latent topics from textual data, which enables the automatic discovery of topics present in the text and reveals their distribution across different documents. This approach does not rely on predefined keywords or co-citation relationships but rather extracts topic information directly from the textual data, enabling more accurate capture of the text's semantic and contextual aspects (Vayansky and Kumar, 2020).

**Figure 1:**
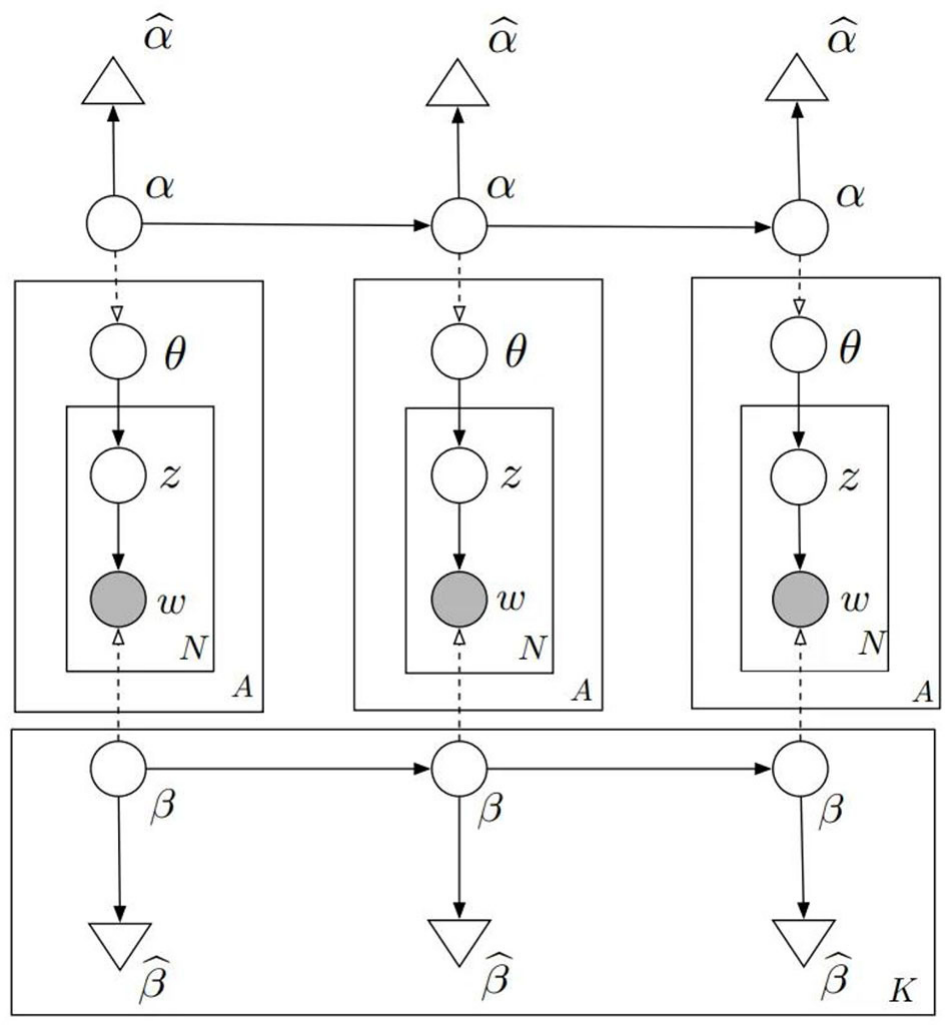
DTM.

Although the classical LDA has been effective at identifying topics, the incorporation of a temporal dimension through the DTM offers a superior capability to trace research topics' developmental trajectory and evolution over time. This temporal analysis is crucial, particularly in fields such as early reading where discourse and thematic emphasis may shift significantly over the years. For instance, the discussion surrounding early reading practices in 1995 is likely to differ substantially from contemporary discourse in 2023. Despite the constancy of the overarching topic of early reading, its nuanced representation and focal areas might evolve. To capture this dynamic nature, we adopted the DTM that Blei and Lafferty (2006) proposed. Building upon the foundation the LDA has laid, the DTM introduces temporal sequencing to the analysis, making it particularly suited to our study, which sought to dynamically identify anticipated developments and transformations within the early reading domain. The practical value of this text mining technique has been widely recognized and applied in the academic community, as evidenced by recent studies (Yao and Wang, 2020; Gao et al., 2022). In our methodology, we began by discretizing and chronologically segmenting the textual data, namely titles, keywords, and abstracts, obtained from the early reading literature. We then hypothesized as to the evolution of topic and content distributions in adjacent time slices, which allowed for the identification of a sequence of continuous topics within the collected abstract data. The specific process of DTM-based topic modeling, which captures these temporal nuances and thematic shifts, is illustrated in Figure 1, where w represents a word, and z denotes the topic to which word w pertains in the collection of documents d within the time slice. Θ follows a Dirichlet distribution with parameter α to determine the topic distribution in the document collection. B is the Dirichlet prior and records the probability of generating a word under a specific topic. The number of words, N, was determined using a Poisson distribution within the time slice. A represents the number of documents within the time slice, and K indicates the number of time slice partitions. For each word in N, the topic z was selected from a multinomial distribution with a parameter value θ, and the word w was chosen based on a multinomial distribution conditioned on z and β. At time t, the topic distribution in the document collection, denoted as α_t_, and the word distribution under each topic, denoted as β_t_, would depend on the previous moment's α_t − 1_ and β_t − 1, *k*_, respectively. The dependency relationships between these were acquired using the dynamic model. The generative process for a sequence of continuous textual documents within the time slice t was as follows:

1. To generate a word distribution: $\beta_{t}|\beta_{t-1}\sim N\left(\beta_{t-\;1,k},\delta^{2}I\right)$
2. To generate a topic distribution: $\alpha_{t}|\alpha_{t-1}\sim N\left(\alpha_{t-\;1},\delta^{2}I\right)$
3. For each document:
  (a) Generate $\eta \sim N\left(\alpha_{t},\alpha^{2}\right)$
  (b) For each word:
  (i) Generate *Z*~*Mult*(π(η))
  (ii) Generate *W*_*t, d, n*_~*Mult*(π(β_*t*_, *z*))

Note that π maps the multinomial natural parameters to the mean parameters. The formula is as follows:


$$\pi (\left(\beta_{k,t}\right)w)=\frac{\exp(\beta_{k,t,w})}{\sum_{w}\exp(\beta_{k,t,w})}$$

In this study, we employed Mimno et al. (2011) concept of “coherence” to determine the optimal number of topics. This involved considering frequently occurring and high-scoring words under a topic and calculating the semantic similarity of their co-occurrence in documents. A higher coherence score indicated stronger model interpretability. The formula is as follows:


$$\begin{array}{l} C\left(k;V^{\left(k\right)}\right)=\overset{M}{\underset{m=2}{\sum }}\overset{m-1}{\underset{l=1}{\sum }}\log\frac{D\left({v_{m}}^{\left(k\right)},{v_{l}}^{\left(k\right)}\right)+1}{D\left({v_{l}}^{\left(k\right)}\right)}\; \end{array}$$

where *D*(*v*) represents the number of documents containing at least one occurrence of word v; *D*(*v, v*′) denotes the number of documents where words v and v′ appear together; and *V*_(*k*)_ = (*V*_(*k*)1_, …, *V*_(*k*)*m*_) represents the list of words most likely to be associated with topic k.

### 3.3 Data collection

To ensure the accuracy and authority of our research sources, we selected Social Sciences Citation Index and Science Citation Index journal articles from the WOS Core Collection to comprise the dataset for this study. The WOS Core Collection is renowned for providing a high-quality literature dataset and is frequently utilized in scientific research (Martín-Martín et al., 2021; Xu et al., 2022). Our search query was constructed as TS = (“early literacy” or “early childhood literacy” or “early reading” or “early childhood reading”), covering the period 1 January 1995 to 31 July 2023, with the latter date being the date of the last search. The year 1995 was selected as the starting point because it is the earliest year available in the WOS Core Collection, as accessed through the Ningbo University Library; hence, the earliest possible retrieval date was set to 1 January 1995. The initial retrieval yielded 1,848 articles. Subsequently, bibliometrics and content analysis were employed to exclude conference abstracts, editorial materials, and other irrelevant types of documents. This refined selection process yielded 1,638 journal articles. Finally, we extracted and synthesized the articles' publication years, titles, abstracts, and keywords to serve as the data source for topic identification.

### 3.4 Analysis of trends in article and citation counts

**Figure 2:**
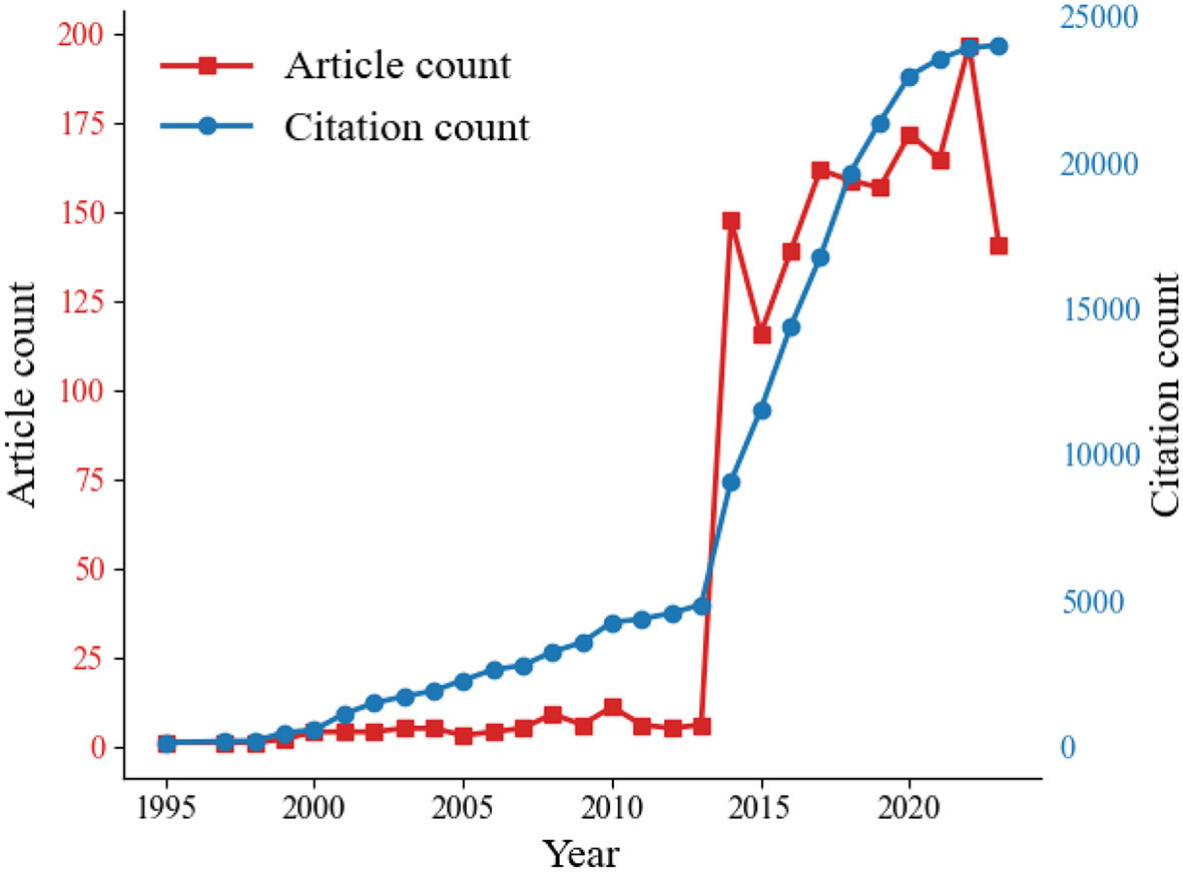
Number of publications and citation counts over the years.

Variation in academic paper quantity over time is an important indicator for assessing development trends in a particular field (Xu et al., 2022). Considering that this study focused on evolution in the early reading field, an occurrence-based statistical method was employed for each time window to obtain the publication volume for each calendar year, as shown in Figure 2, where the red line represents the changes in publication volume in early reading over the years, and the blue line represents the cumulative citation count of publications within each time window. Overall, the total annual publication volume showed a general increasing trend, with the final year's statistics differing from those of the initial year by a factor of over 100. Starting from 2014, there has been a rapid increase in the annual publication volume in the later period of the timeline, indicating significant growth in early reading research in recent years. Furthermore, upon closer examination of the distribution pattern of the cumulative citation count in Figure 2, a consistent year-by-year increase can be observed in the citation frequency of research achievements in early reading, with nearly 25,000 citations by 2023. This demonstrates the increasing influence of early reading research within the academic community. However, the slight decrease in the data for the year 2023 should be noted, which may be attributed to the choice of the retrieval time point in this study.

### 3.5 Data cleaning and preprocessing

Prior to engaging in topic modeling, it is imperative to enhance the quality of the data through rigorous preprocessing. This foundational step ensures that the subsequent optimal topic identification is both accurate and insightful. Data preprocessing in this study entailed the following steps.

Step 1 entailed novel word discovery and synonymous substitution. In early reading research, we often encounter terms that intersect with complex fields such as neuroscience. Such terminology is typically intricate and frequently presented in abbreviated forms, such as “executive function (EF).” In this study, we meticulously identified and tagged these specialized terms first to ensure that they could be accurately recognized and processed in tokenization and handling, thereby preserving their original academic significance. Following the identification and tagging process, we proceeded with synonymous substitutions. To maintain the integrity of the original semantic meanings, we replaced certain longer terms with their widely accepted abbreviations; for example, we substituted “functional magnetic resonance imaging” with “fMRI,” “deafness or hard of hearing” with “DHH,” and “speech sound disorders” with “SSD.” Consequently, even after tokenization and processing, the nuanced meanings of these terms were retained and emphasized.

Step 2 entailed tokenization, stop-word removal, and lemmatization. Before applying the DTM for topic modeling, the text must first be tokenized to construct a vocabulary. Following tokenization, we filtered out non-informative stop words to refine the dataset. Subsequently, utilizing the Natural Language Toolkit library's lemmatize method, we performed lemmatization to restore the words to their base or dictionary form, focusing primarily on nouns and adjectives. This step was crucial for reducing the data's complexity and enhancing interpretability.

Step 3 entailed text vectorization, which is the process of converting text into numerical values to facilitate computational analysis. In this study, we employed the Gensim library's “corpora” module. We adopted the bag-of-words model and represented the tokenized text as frequency vectors. This transformation was pivotal for applying mathematical and statistical operations in the subsequent topic modeling phase.

By meticulously executing these preprocessing steps, we ensured a robust and clean dataset and laid the groundwork for effective optimal topic identification. This sequential approach not only enhanced the study's academic rigor but also ensured a seamless transition from data preparation to in-depth topic analysis, which culminated in a comprehensive understanding of the underlying textual topics.

### 3.6 Optimal topic identification

**Figure 3:**
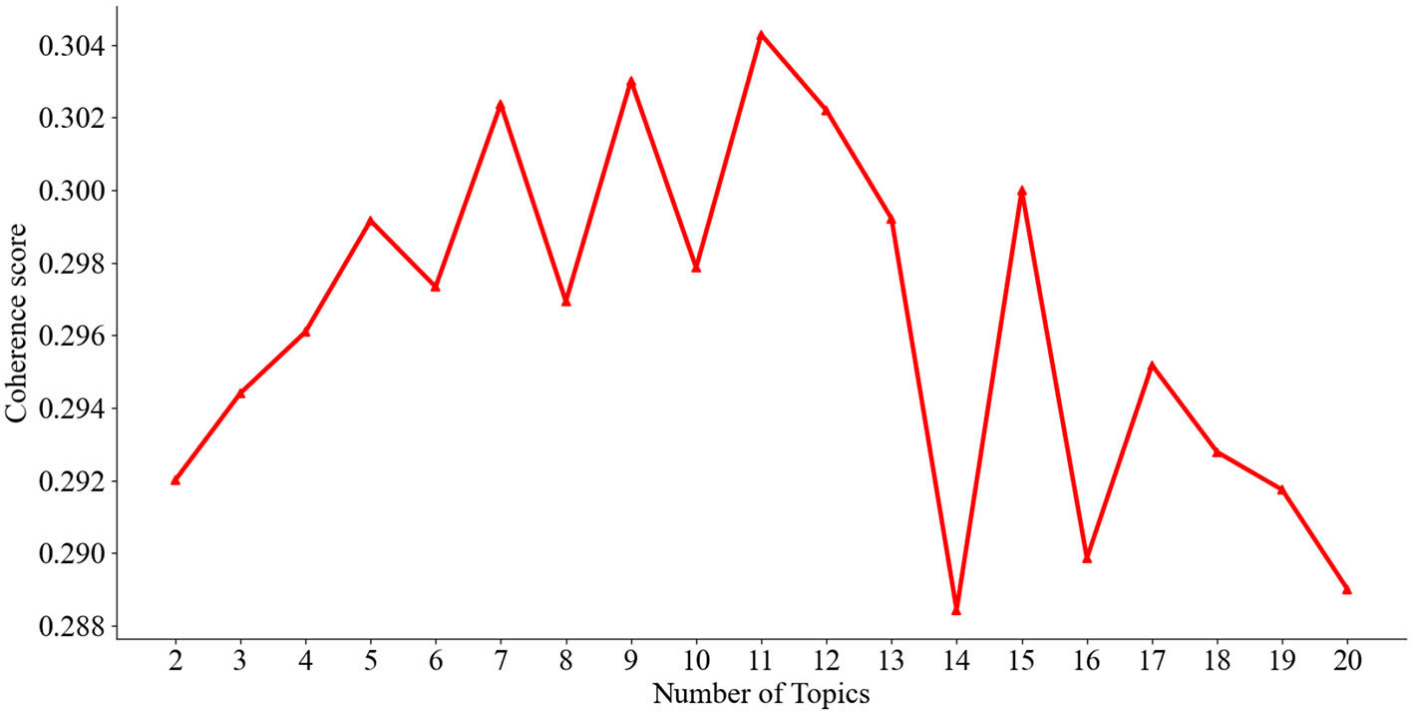
Coherence scores.

In topic modeling, determining the optimal number of topics is an important and challenging task. This study identified the ideal number of topics by accurately calculating the model's coherence. Specifically, we set the topic number range as 1–20 and iterated the calculation in steps of 2. The coherence score, ranging between 0 and 1, indicated the quality of topic segregation, with a higher score representing better quality. The topic coherence results are presented in Figure 3. When the number of topics was set to 11, the model exhibited the highest coherence score, leading us to determine that the optimal number of topics was 11. We further designated that each topic would contain ten primary terms (Table 1). Utilizing the multidimensional mapping relationship between the literature topics and the topic terms, we conducted a comprehensive analysis of different reading topics and their corresponding topic terms for each sub-period. This method allowed us to track and analyze the evolution of topics with fine granularity, which resulted in insights into understanding the text.

**Table 1:** Topic–word mapping within each time period (partial).

**Topic**	**(Time = 1995)……(Time = 2022)**	**(Time = 2023)**
Topic 1	**…**	Literacy, child, reading, early, teacher, read, student, school, book, intervention
Topic 2	**…**	Awareness, phonological, word, Chinese, reading, read, letter, study, vocabulary, child
Topic 3	**…**	Achievement, mathematic, EF, early, academic, executive, skill, reading, mediate, child
Topic 4	**…**	Writing, write, skill, child, early, letter, preschool, literacy, composition, teacher
Topic 5	**…**	Language, child, English, literacy, Spanish, bilingual, skill, early, study, use
Topic 6	**…**	Library, family, public, story time, book, program, librarian, literacy, child, community
Topic 7	**…**	Speech, SSD, sound, production, phonological, child, age, auditory, DHH, difficulty
Topic 8	**…**	Dyslexia, reading, read, child, risk, word, phonological, awareness, early, development
Topic 9	**…**	Genotype, imputation, study, data, genetic, reading, ability, twin, longitudinal, variable
Topic 10	**…**	Synapse, energy, spike, SNN, device, stochastic, hardware, VOT, SOT, visual
Topic 11	**…**	Child, autism, early, spectrum, skill, age, study, parent, school, ASD

## 4 Results

### 4.1 Topic content analysis

**Figure 4:**
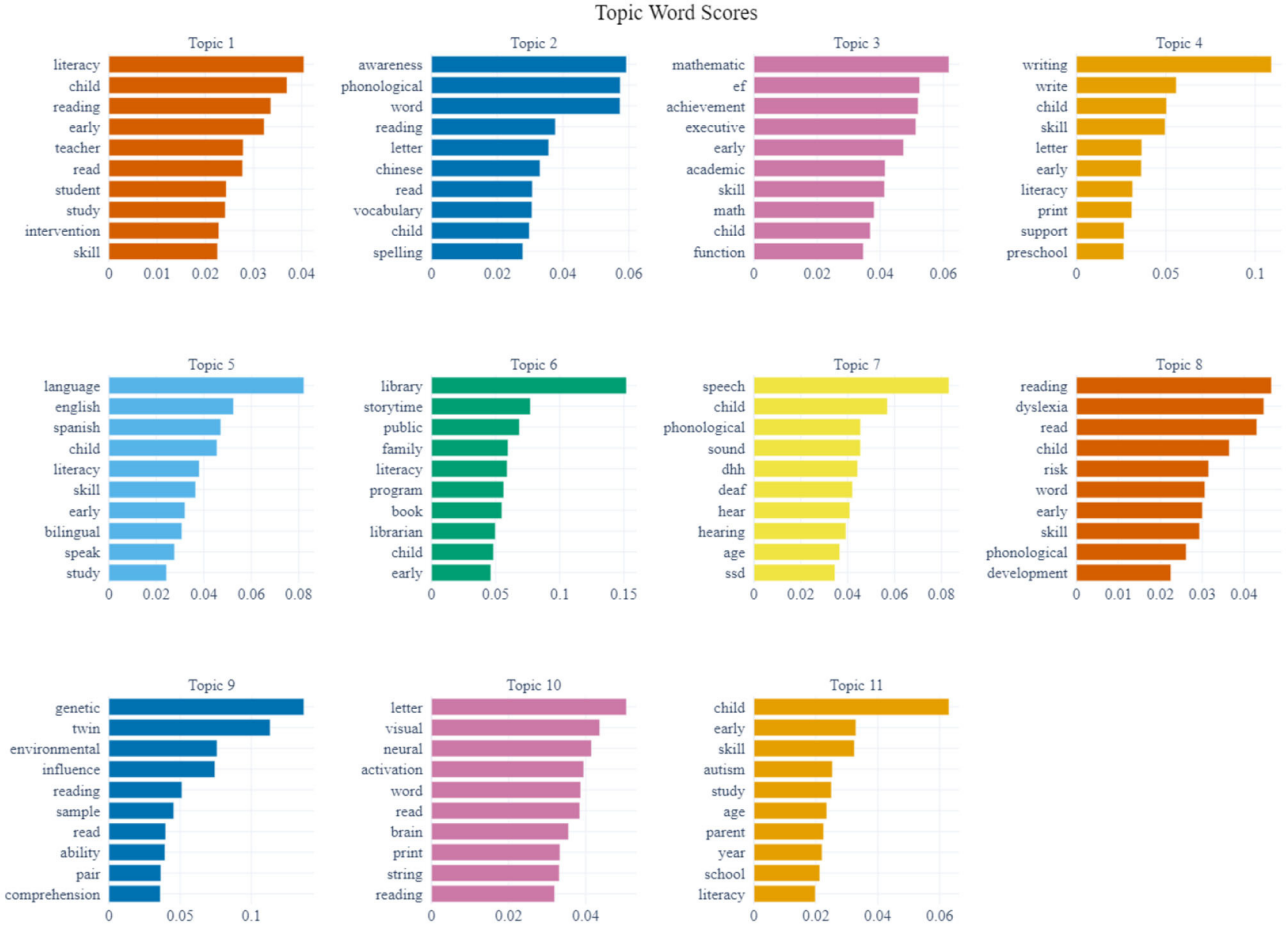
Visualization of topic–word distribution.

As shown in Figure 4, we visually displayed the top ten feature words for each of the 11 topics. This visualization aided in our understanding and analysis of the corresponding topic content. Additionally, we selected representative literature from the document–topic distribution graph to provide an in-depth analysis of topic content. The results by topic are as follows.

#### 4.1.1 Topic 1: Interventions' impacts on early reading competencies

Under this topic, key terms like “literacy,” “child,” “reading,” “early,” “teacher,” “read,” “student,” “study,” “intervention,” and “skill” emerged as especially representative. The breadth of academic inquiry in this area spans the effectiveness of various instructional strategies to the significance of the home literacy environment (HLE). Key considerations include the design and implementation of teacher-led interventions (Carta et al., 2014; McConnell et al., 2014), the continuous evolution of these strategies to keep pace with changing educational landscapes (Kaminski and Powell-Smith, 2017; Phillips et al., 2021), and dynamic evaluations regarding the success and challenges of such interventions (Gustafson et al., 2014; Greenwood et al., 2017). Within this overarching topic, three primary facets stood out.

Firstly, teacher interventions in early reading play a pivotal role in early reading development. In environments with limited resources and diverse linguistic backgrounds in particular, the strategies teachers employ are crucial. Comprehensive literacy guidance in early reading classrooms can greatly enhance students' literacy skills. For instance, Putman (2017) highlighted the impact of teacher support on preschool literacy achievements. Similarly, Mihai and van Staden (2019) emphasized educators' vital roles and noted the varied effectiveness of strategies across different cultural and resource settings. It is therefore imperative to consider both cultural and resource disparities when designing these strategies.

Secondly, the HLE plays a central role in early reading development. Sénéchal and LeFevre (2014) conducted a 2-year longitudinal study involving 110 children that delved deep into the continuous influence of and changes in the HLE on the children's early vocabulary and reading abilities at different junctures. The research findings revealed that stability and variations in the HLE serve as key predictors for early vocabulary and reading growth. Further supporting this point, Zhang S.-Z. et al. (2023) evaluated 553 Chinese kindergarten third-graders and examined family factors to investigate the direct and indirect effects of the family reading environment on the children's early literacy skills. The research results demonstrated that the family reading environment both directly and indirectly influences early literacy skills. Moreover, the differences in these influences between affluent and impoverished societies were found to be minimal, suggesting the universality of the family reading environment's impact on early literacy abilities. These research findings have provided valuable insights for the development of educational strategies and family support programs aimed at promoting early literacy skills.

Thirdly, regarding targeted reading interventions, in addition to traditional early reading interventions, there has been significant scholarly interest in specialized child populations with reading disabilities, and researchers have proposed specific intervention methods to enhance early literacy skills in these specialized groups (Pace Miles et al., 2019). For a salient example, consider the tailored interventions designed for children with Down syndrome. Studies have evaluated the feasibility and potential impacts of these bespoke interventions and reported promising outcomes (Lemons et al., 2017). Such focused research underscores the importance of customizing strategies to cater to distinct needs within varied child populations. This brings a fundamental question to the forefront: How should interventions be tailored to meet specific requirements? To address early reading challenges, strategies should not only be culturally sensitive but also tailored to individual needs. Consistent collaboration between families and educational institutions is imperative in this process. This synergistic approach ensures that reading interventions are both continuous and consistent, thus creating a comprehensive learning environment for children.

#### 4.1.2 Topic 2: Foundational elements in early reading: phonological awareness, letters, and spelling

Reading is the cornerstone of early learning and cognitive development, a status that renders understanding its key components crucial for education. The core terms under Topic 2, such as “awareness,” “phonological,” “word,” “reading,” “letter,” “Chinese,” “read,” “vocabulary,” “child,” and “spelling,” have illuminated an inherent nexus between preschool children's reading proficiency and their phonological awareness, grasp of the alphabetic principle, and orthographic knowledge. Assiduous ongoing scholarly scrutiny in this domain seeks to elucidate the determinants shaping precocious reading aptitudes.

Regarding phonological awareness, Diamanti et al. (2017) evaluated 104 preschoolers' early phonological awareness skills and researched its predictive power on reading and spelling outcomes at the end of Grade 1. Those scholars found that phonological awareness played a vital role in assessing the preschoolers' fluency in reading text as well as their comprehension of the text. These findings align with those of Kargiotidis et al. (2023), who administered early reading development tests to 141 first-graders to examine the impact of their vocabulary knowledge, phonological awareness, and morphological awareness on their early reading skills and Greek spelling consistency and found that phonological awareness is a significant predictor of word reading accuracy.

Regarding letter knowledge, Plewko et al. (2018) proposed that learning letter–sound (LS) association, or letter knowledge, is a significant determinant of reading ability. However, whether the disruption of LS association leads to reading impediments remains uncertain. To explore this, those scholars employed functional magnetic resonance imaging (fMRI) to probe the relationship between LS association and reading proficiency and found that variations in LS association can predict future reading issues in children. This finding complements those of Kuvač-Kraljević et al. (2019), who conducted a cross-sectional study involving 764 children to delve into the interrelation and inherent structure of phonological awareness and letter knowledge during the early literacy phase. Their findings have proffered valuable insights for educational policies in the realm of early literacy.

Focusing on spelling, Ouellette and Sénéchal (2017) examined the “invented spelling” abilities of 117 children, positing it as a predictor of subsequent reading and spelling competencies in Grade 1. Their research has indicated that the invented spelling approach influences children's subsequent reading capabilities and explains variations in their literacy outcomes. Additionally, Ye et al. (2022b) longitudinal study found an interconnection of early spelling and reading abilities in children transitioning from Cantonese kindergartens in Hong Kong to primary schools, with early spelling and reading performances influencing each other.

In summary, phonological awareness, alphabetic knowledge, and spelling are undoubtedly the foundational pillars of early reading. Although numerous studies have examined these elements individually and sometimes in tandem, a holistic understanding of their dynamic interplay, especially in multilingual contexts, remains relatively underexplored. Notably, the intricate relationships among these components, as they manifest across diverse linguistic landscapes, might offer vast research potential.

#### 4.1.3 Topic 3: Early mathematical ability and reading achievement

Within the purview of Topic 3, pivotal terms such as “mathematic,” “EF,” “achievement,” “executive,” “early,” “academic,” “skill,” “math,” “child,” and “function” underscored the complex intersectionality between early mathematical competencies and reading milestones. Of particular intrigue is the modulatory role executive functions (EFs) seem to play in shaping academic outcomes. Although there exists a consensus on the synchronicity between mathematical and reading proficiencies, the nuanced pathways through which the former informs the latter, especially in diverse linguistic or pedagogical contexts, have not yet been comprehensively delineated.

Venturing into this relatively uncharted domain, Purpura et al. (2017a) assessed 125 preschoolers' mathematical literacy and cognitive landscapes to distill the mechanisms underpinning how early mathematical prowess might serve as a prognosticator for emergent literacy milestones. Their analysis unveiled a nuanced interface between early mathematical literacy and reading proficiencies, accentuating the mediating role of mathematical language skills therein. To better understand the nexus between early mathematical abilities and early reading skills, Purpura et al. (2017b) extrapolated how EFs' multifaceted elements interweave with math and reading skillsets in preschoolers. Their illuminative outcomes not only demystified the intricate interrelationships but also spotlighted the anticipatory weight of early math skills vis-à-vis reading evolution. Additionally, Ten Braak et al. (2022) further substantiated EFs' significance in elucidating the relationship between toddlers' early math skills and their elementary school mathematical and reading achievements. In summary, the intricate tapestry that interlinks early mathematical cognition, reading attainment, and EFs remains a profound area for academic exploration.

#### 4.1.4 Topic 4: Supporting early literacy skills and fostering preschoolers' writing abilities

Central keywords under Topic 4 were “writing,” “write,” “child,” “skill,” “letter,” “early,” “literacy,” “print,” “support,” and “preschool.” These terms highlighted scholarly interest in preschool-aged children's writing abilities. Although early writing proficiency is a crucial component of literacy development, limited research has been conducted on promoting its growth in early educational settings, despite recent studies and policy reports underscoring the significance of early writing support. Nonetheless, some scholars have delved into this area.

For instance, Gerde et al. (2015) focused on establishing a reliable and effective set of instructional standards for preschool writing skills and utilized these criteria to evaluate teaching practices in writing and their correlation with early writing performance. Bingham et al. (2017) further explored the subject, emphasizing how educators support child writing in classrooms and the relationship between this support and child writing development. Their findings have indicated that educators frequently concentrate on early handwriting and spelling, often sidelining writing content. However, children whose writing received attention demonstrated enhanced writing skills, a finding that has offered insight into potential instructional strategies and policy considerations. As Bingham et al. (2017) pointed out, although focusing on handwriting and spelling predominates, this does not negate the importance of studying other aspects of early writing. In response, Quinn et al. (2021) voiced some criticisms, noting that recent research on preschool writing skills, although increased in quantity, still leans excessively toward handwriting and spelling. Consequently, those scholars examined preschoolers' compositional abilities to identify the various factors influencing their performance. The study found correlations between early compositional capabilities, writing complexity, reading proficiency, and cognitive skills and has offered valuable insights into understanding early creative abilities and their relationship with early literacy skills.

In summary, although the scaffolding of handwriting and spelling remains an undeniable focal point in teaching, the broad realm of early writing in preschool-aged children, intertwined with creativity, cognition, and literacy skills, calls for more comprehensive, in-depth academic reflection and pedagogical innovation to support a robust future literacy base.

#### 4.1.5 Topic 5: Cultivating early reading skills in bilingual children at the intersection of the Spanish and English linguistic spheres

Under Topic 5, the top ten keywords were “language,” “English,” “Spanish,” “child,” “literacy,” “skill,” “early,” “bilingual,” “speak,” and “study,” suggesting that the central focus of this topic has been bilingual children's early reading abilities, particularly in the context of Spanish–English bilingualism. The intellectual dialogue surrounding this domain is notably augmented by Cummins (1981) influential common underlying proficiency model, which postulates a linguistic synergy, indicating that when children acquire academic knowledge and skills in their native language, they concurrently obtain language-independent information that can be applied when learning a second language. Supported by this theoretical framework, Goodrich et al. (2016) conducted an empirical study involving 554 native Spanish-speaker children and further endorsed Cummins' (1981) assertion with findings indicating that reading skills in a child's native language indeed positively influence their acquisition of writing skills in a second language. In this regard, Goodrich and Lonigan (2017) utilized confirmatory factor analysis to evaluate the common underlying proficiency model's relevance to the early literacy skills of 858 preschoolers whose native tongue was Spanish and found that the preschoolers shared a set of foundational competencies encompassing both language-related and language-independent skills. This finding suggests that foundational abilities such as reading skills can traverse distinct languages and facilitate knowledge transfer. These results have provided more tangible empirical support for Cummins' (1981) model. However, language learning is not solely influenced by intrinsic linguistic factors. Wackerle-Hollman et al. (2022) studied 313 Spanish–English bilingual preschoolers and found that both cultural aspects and classroom language instruction methods are correlated with early reading performance. In summary, although the common underlying proficiency model offers a robust scaffolding for interpreting bilingual literacy, it is imperative to appreciate that early reading in bilingual contexts is a confluence of both intrinsic linguistic cadences and extrinsic pedagogical and cultural nuances.

#### 4.1.6 Topic 6: Building an early reading environment for young children involving public libraries, the family, and the community

Key terms under Topic 6, such as “library,” “story time,” “public,” “family,” “literacy,” “program,” “book,” “librarian,” “child,” and “early,” underscored public libraries' pivotal role in fostering early literacy environments, as well as the significance of community outreach programs. Under this topic, scholars have focused on strategies to cultivate an environment conducive to early childhood literacy development. For instance, Kociubuk and Campana (2019) found that current story time activities predominantly employ narrative-driven books and feature fewer expository and informational stories. They have advocated for diversifying story genres in public libraries and incorporating contemporary narratives to bolster children's early literacy development. Furthermore, Cahill et al. (2020) found a strong correlation between parents' willingness to participate in library story time and their children's interest in reading. Such findings have presented compelling evidence to enhance the early reading environment and kindle children's interest in reading by emphasizing collaboration between communities and families.

#### 4.1.7 Topic 7: Early phonological awareness training for children with deafness or hard of hearing and/or speech sound disorders

Children with hearing impairments face substantial challenges in acquiring early phonological awareness, which can have profound implications for their subsequent reading abilities. Central terms under Topic 7, such as “speech,” “child,” “phonological,” “sound,” “DHH,” “deaf,” “hear,” “hearing,” “age,” and “SSD,” highlighted the intricate relationship between these children's phonological awareness and early reading skills development.

Cupples et al. (2014) seminal study found that children with deafness or hard of hearing (DHH) who received an early cochlear implantation displayed heightened phonological discernment, a potential harbinger of their evolving reading proficiencies. Interestingly, this research elucidated a spectrum of convergences between DHH children and those with normal hearing and has bridged domains such as phonological prowess, alphabetic cognition, and lexicon acquisition. Pedagogical innovations aimed at improving this cohort's literacy outcomes have come to the forefront. Lederberg et al. (2014) pivotal exploration has attested to the efficacy of certain tailored educational strategies in nurturing reading proficiencies among DHH children. Parallelly, speech sound disorders (SSD) loom large and have been marked as potential crucibles for impediments in reading and phonological synthesis. Navigating this complex topography, Tambyraja et al. (2020) analytically assessed a cohort of young students spanning kindergarten to Grade 2 to map the risk contours associated with SSD vis-à-vis reading adversities and decipher the intricate web interlinking these risk profiles with reading hurdles.

In summary, as we traversed the multifaceted landscape of early phonological awareness in children with DHH and SSD, it became manifestly clear that their literacy pathways, though riddled with challenges, are also replete with possibilities. This dynamic interplay beckons a deeper, more nuanced academic introspection and proactive pedagogical recalibration to facilitate optimized literacy trajectories for these young children.

#### 4.1.8 Topic 8: Disentangling early dyslexia through proactive detection and the identification of neurobiological underpinnings

Keywords under Topic 8 were “reading,” “dyslexia,” “read,” “child,” “risk,” “word,” “early,” “skill,” “phonological,” and “development,” indicating that the topic centers around the early identification of reading dyslexia in preschoolers. Several studies have provided valuable insights, including Saygin et al. (2013), who initially attracted attention for employing neuroimaging techniques to investigate white matter development in children at risk for familial dyslexia. Surprisingly, they discovered a close relationship between white matter development and reading abilities, particularly the integrity of the left arcuate fasciculus white matter structure, which plays a crucial role in reading ability. This finding has provided a neurobiological basis for predicting early reading difficulties.

Powers et al. (2016) further explored the relationship between preschoolers at risk for familial dyslexia and their family cultural environment, phonological processing, and neural activation. Those scholars' findings revealed significant differences in family environment and neural activation between children at genetic risk for dyslexia and typically developing children. This not only confirmed Saygin et al.'s (2013) findings but also emphasized the importance of early intervention and family support in preventing reading difficulties. Kraft et al. (2016) addressed the challenge of accurate prediction and early intervention by combining cognitive measurements and neuroimaging techniques in an attempt to identify early markers of reading difficulties in preschoolers. Those scholars concluded that such an approach significantly improves the accuracy of predictively identifying children at risk for dyslexia, and their research has provided a powerful tool for developing early intervention measures that could alleviate the impact of reading dyslexia. In essence, the multifaceted research on early dyslexia underscores the paramount importance of combining advanced neurobiological insights with proactive pedagogical strategies to produce optimal child reading outcomes.

#### 4.1.9 Topic 9: Interactive factors in early reading: genetics and the environment

Individual differences in early reading abilities are associated with the interplay between genetic and environmental factors. Key terms under Topic 9, such as “genetic,” “twin,” “environmental,” “influence,” “reading,” “sample,” “read,” “ability,” “pair,” and “comprehension,” indicated that scholars have actively explored the impacts of genetics and the environment on the development of early reading abilities. Several pivotal studies have offered insightful perspectives on that relationship. Firstly, Taylor and Schatschneider (2010) examined a sample comprising 1,401 kindergarten twin pairs and 1,285 elementary school twin pairs aged 5–7 years, controlling for factors such as family income and ethnicity. Their findings have suggested a potential association between socioeconomic environment and the etiological structure of early literacy abilities. Building on previous studies, Petrill et al. (2010) analyzed latent growth curve models derived from measurements of 314 twin pairs participating in Western Ohio's preparatory reading program. Those scholars focused on the relationships between reading level growth rates at different developmental stages and genetic and environmental factors. Furthermore, Schenker and Petrill (2015) employed both univariate and multivariate quantitative genetic models to measure data obtained from 284 pairs of children with an average age of 9.81 years. Their study found a strong correlation between reading motivation and reading ability in the presence of non-shared environmental factors. Collectively, these investigations have underscored the importance of the interaction between genetics and the environment in the development of early reading abilities and have provided valuable insights into the mechanisms underlying individual differences.

#### 4.1.10 Topic 10: Early reading development: visual and neural activation

Under Topic 10, core keywords such as “letter,” “visual,” “neural,” “activation,” “word,” “read,” “brain,” “print,” “string,” and “reading” underscored researchers' commitment to examining the intricate relationships among specific visual regions, neural activations, and early reading development. Gaillard et al. (2003) employed fMRI and echo-planar imaging techniques to investigate the neural network patterns associated with cognitive development in 16 native English-speaking children with an average age of 7.2 years. Notably, the findings highlighted pronounced activity in the left temporal–occipital junction, middle frontal gyrus, and supplementary motor area, suggesting that in children aged 6–7 years, the neural networks responsible for reading processes exhibit significant lateralization and region-specificity. Gaillard et al.'s (2003) research paved the way for further investigations and enriched our understanding of neural activations in early reading development. Maurer et al. (2005) further enriched the understanding of the connection between specialization for letter strings and early literacy skills. The study found that printed word letter strings activate specific visual areas in the brain. Based on neural physiological recordings of brain activity, this response was hypothesized to be a form of plasticity change, underscoring the close link between early literacy skills and letter string specialization. Furthering the narrative, Malins et al. (2018) harnessed fMRI techniques to delve deeper into the nexus between individual reading capabilities and variabilities in neural activations within reading networks. Their findings have shown that inter-trial variations in neural activation positively impact an individual's reading prowess, thus offering fresh insights into neural plasticity in early reading development. Cumulatively, these investigations have reinforced the intertwined relationships among visual perception, neural activation, and reading skills and have provided substantial backing for a profound understanding of early reading evolution.

#### 4.1.11 Topic 11: Early literacy proficiencies in children with ASD

Topic 11 encompassed popular keywords such as “child,” “early,” “skill,” “autism,” “study,” “age,” “parent,” “year,” “school,” and “literacy,” indicating a focus on analysis of literacy skills in children with early ASD that has yielded crucial information for future interventions as well as support for early literacy abilities in children with ASD. For instance, Davidson and Ellis Weismer (2014) examined the characteristics and predictive factors of early reading abilities in 152 children with ASD and emphasized the significance of early language skills in their development of reading abilities. Solari et al. (2022) analyzed the development of early reading skills in 616 preschool-aged children who had been diagnosed with ASD educationally and found heterogeneity in their early preschool-level reading skills. These research findings have important implications for early educational interventions and literacy education for children with ASD in preschool settings.

Furthermore, Kiliç-Tülü et al. (2023) conducted a comparative study on the early literacy skills of children with ASD and those of typically developing children, with a focus on the influence of the HLE in the Turkish language context. The study revealed the characteristics of early literacy skills development in children with ASD with a Turkish language background and highlighted the significant predictive role of the HLE, non-verbal cognitive abilities, and working memory. The study further emphasized the importance of effectively teaching early literacy skills to children with ASD. In essence, although children with ASD may traverse unique developmental paths, the interplay of the HLE, cognitive faculties, and linguistic foundations profoundly shapes their early literacy narratives.

### 4.2 Analysis of topic popularity and the evolution of core keywords

**Figure 5:**
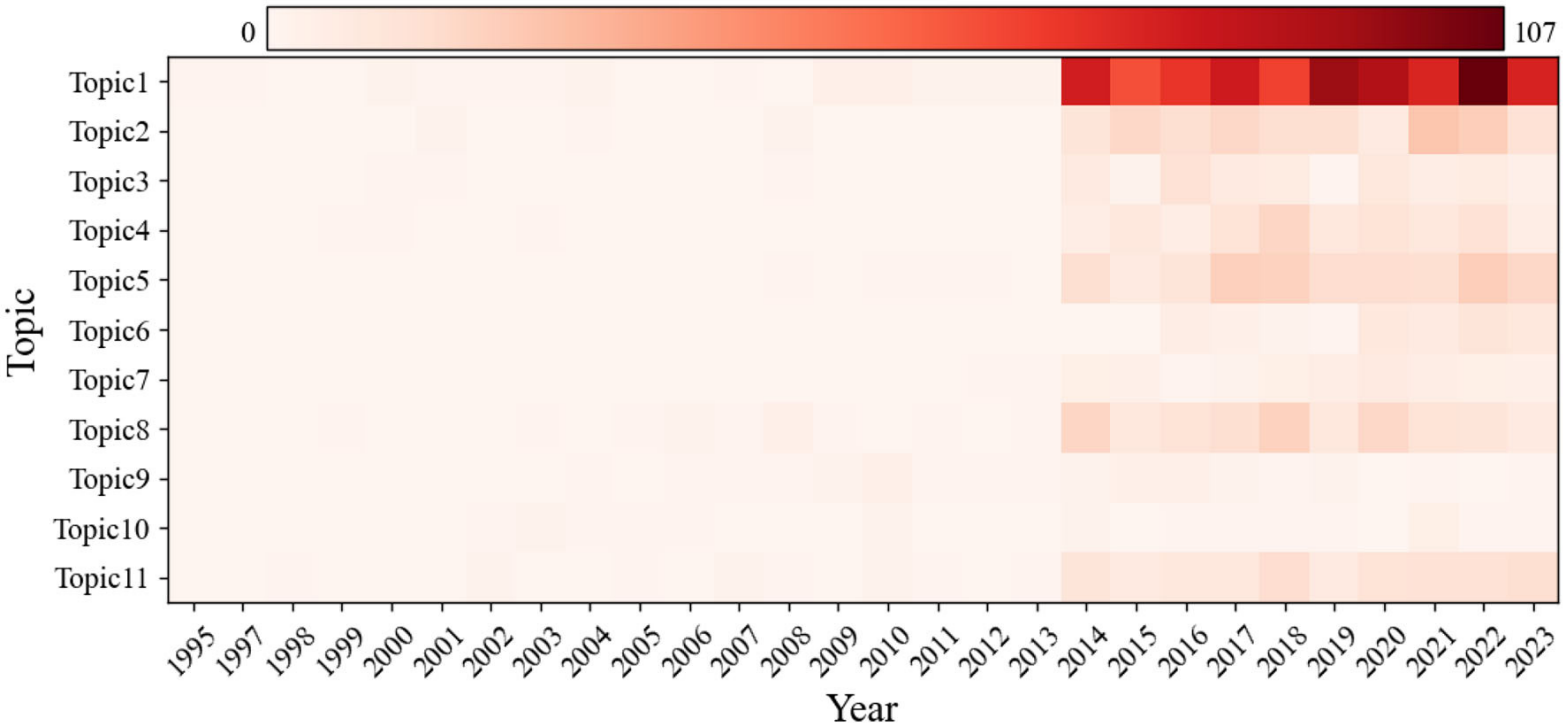
Topic evolution heatmap.

As time progresses, the research focus within the same topic undergoes significant changes at different stages of development. To gain a deeper understanding of this evolutionary process, this study utilized the DTM to comprehensively analyze thematic evolution and trends in the field of early reading. We have identified and presented the document distribution characteristics of each topic over different time periods, as depicted in Figure 5. We also obtained a topic–word matrix for each stage, as shown in Table 1. By analyzing the top ten keywords with the highest occurrence probability at each stage, we clearly delineated the evolution of research focuses over time. This not only aids in our understanding of past research trends but also provides a solid foundation for predicting future research directions.

We found consistently increasing research interest in Topics 1, 2, 5, 6, and 11. Topic 1 in particular has always been a prominent research hotspot in early reading. Although an overall trend of increased research interest was found for the remaining four topics, changes have been relatively moderate. The keyword evolution analysis showed that “literacy,” “children,” “early,” “reading,” and “teacher” have always constituted the core of Topic 1, highlighting early childhood reading's significance and educators' crucial role (Snell et al., 2022). However, recent trends have revealed a notable shift within Topic 1, characterized by the increased frequency and prominence of the keyword “book.” Accompanied by an escalating focus on shared book reading (Miller-Goldwater et al., 2022; Shen and Del Tufo, 2022), picture books (Shimek, 2021; Zhang R. et al., 2023), and digital books (Kim, 2022; Korat et al., 2022), this change has indicated a paradigmatic transition from traditional reading strategies toward a more integrated approach that embraces modern digital tools and methodologies in educational practices, reflecting a broader shift in pedagogical paradigms and research interests within the field. Under Topic 2, the addition of the keywords “Chinese” and “vocabulary” reflects growing emphasis on early reading in the Chinese linguistic context (Hemelstrand et al., 2023; Lin and Zhang, 2023). Although we found increased research interest in Topic 5, the evolution of its keywords did not demonstrate significant new trends or shifts, suggesting that research in this area may still be focused on longstanding core issues or that fresh perspectives and innovations may be needed to stimulate new momentum and directions. Results for Topic 6 revealed recent research attention to “family,” “community,” and “partnerships,” highlighting families' and communities' roles in early reading (Gillanders and Barak, 2022; Smith et al., 2023). The latest hotspots under Topic 11 were identified as “parents,” “teachers,” and “schools,” indicating increasing interest in families' impact on early skills development among children with ASD (Gasamis et al., 2023) and teacher-driven early in-school reading interventions for children with ASD (Macdonald et al., 2022). Notably, Topics 11 and 1 are highly interconnected, and their potential combined future trajectory might become a central focus in the field of early reading.

Conversely, Topics 7, 9, and 10 have exhibited a downward trend in research interest. Recent changes in publication volume reflect limited research outcomes for these topics, suggesting declining academic focus. Without new research perspectives or opportunities, interest in these topics is likely to continue diminishing. Moreover, the keyword evolution analysis did not identify any emergent research foci in these topics in recent years.

Topics 3, 4, and 8 exhibited relatively stable trends. Although Topic 4 showed a slow upward trajectory, the overall increase was modest, indicating a stable overall pattern. This suggests that although these topics may have some research basis, the likelihood of them gaining further attention and becoming hot topics in the future is relatively low. The keyword evolution analysis showed contemporary research interest under Topic 3 in “reading,” “media,” and “children,” emphasizing the media's role in early reading. Under Topic 4, we found increased focus on “early childhood education,” “composition,” and “teachers,” indicating the growing recognition of the importance of early education and the teacher's role (Rylak et al., 2022; Tortorelli et al., 2022). Additionally, under Topic 8, increasing interest in “phonological awareness,” “vocabulary,” and “reading” has highlighted growing attention to cognitive linguistic processes in individuals with reading disorders. The similarity with Topic 2 suggests a potential joint trajectory for future research.

In summary, this comprehensive analysis revealed the document distribution characteristics of each topic across different time periods and identified the continuously evolving research focuses. Although the keyword evolution of certain topics, such as Topic 5, did not show significant new trends or shifts, these results highlight the need for new perspectives and innovation to drive development in those areas.

## 5 Discussion

Utilizing literary data obtained from the WOS Core Collection, this study delved into the textual data of topics related to early reading using the DTM and visually analyzed the characteristics and evolutionary patterns of early reading topics. From a quantitative perspective, this study interpreted the characteristics and evolutionary trends of academic attention to early reading and derived the following main findings.

Firstly, academic discussions on early reading have primarily focused on 11 major topics: interventions' impacts on early reading competencies (Topic 1); foundational elements in early reading: phonological awareness, letters, and spelling (Topic 2); early mathematical ability and reading achievement (Topic 3); supporting early literacy skills and fostering preschoolers' writing abilities (Topic 4); cultivating early reading skills in bilingual children at the intersection of the Spanish and English linguistic spheres (Topic 5); building an early reading environment for young children involving public libraries, the family, and the community (Topic 6); early phonological awareness training for young children with DHH and SSD (Topic 7); disentangling early dyslexia through proactive detection and the identification of neurobiological underpinnings (Topic 8); interactive factors in early reading: genetics and the environment (Topic 9); early reading development: visual and neural activation (Topic 10); and early literacy proficiencies in children with ASD (Topic 11). Among these, Topics 1, 2, 5, 6, and 11 have received particular attention, which reflects their significant standing in the field. In contrast, Topics 7, 9, and 10 have seen limited research outputs in recent years, suggesting a possible shift in research focus or a need for new perspectives and innovations in these areas.

Secondly, as time progresses, individual topics exhibit a unique developmental trajectory and dynamic changes. Time-series analysis of core thematic words revealed varying degrees of stability, with some key terms such as “literacy” and “reading” remaining stable, whereas emerging keywords like “book” have begun to make significant appearances under Topic 1, indicating an ongoing evolution of research perspectives and foci. This is exemplified in the results of analyzing the evolution of core keywords under Topic 1, as reported in Section 5.2 (see Table 1). Specifically, the results have highlighted the growing importance of modern digital tools in early childhood reading interventions. Moreover, computational analysis has revealed emerging research frontiers across various topics, providing valuable insights for scholars, policymakers, and practitioners in the field of early reading. This not only deepens the understanding of the knowledge structure and evolutionary patterns within the field but also emphasizes the importance of comprehensively grasping early childhood reading developmental trends by capturing these dynamic changes.

### 5.1 Implications of the study

#### 5.1.1 Theoretical implications

This study is the first comprehensive quantitative analysis of the early childhood reading field using the DTM for text mining. It thoroughly assessed the current state and developmental trends of research in this area. Theoretically, this study is distinguished by the following features.

Firstly, the study is notable for its objectivity and innovative research methodology. Unlike traditional systematic reviews in the field, this study was grounded in quantitative analysis via text mining, which significantly reduced subjective bias and ensured the objectivity and replicability of the results. The study's demonstration of such an innovative approach provides a new perspective and a scientific tool for exploring the knowledge structure in the field of early childhood reading more precisely and scientifically.

Secondly, this paper comprehensively discusses intervention strategies. The study delved into various aspects of early reading interventions, including teacher interventions, the HLE, and customized educational plans for children with special needs. The findings underscore the necessity of considering the interplay of educational settings, family backgrounds, and individual differences in children for successful early reading interventions. This provides new theoretical support for designing effective early reading intervention strategies in diverse cultural and economic contexts.

Thirdly, the research has expanded cross-cultural and bilingual reading theories. By analyzing bilingual children's early reading abilities, this research has revealed the complexity of language skill interactions in bilingual environments, an insight that is significant for developing cross-cultural and bilingual reading theories, especially in non-English-dominant linguistic contexts. Hence, the study offers educators and researchers a critical framework for assessing and enhancing early reading abilities in bilingual children.

Fourthly, this study draws attention to applying neurobiology to identify early reading disorders. The study highlights the importance of understanding the neurobiological underpinnings of early reading disorders, such as dyslexia. For early detection of familial reading disorders in particular, these findings provide new directions for future research using neurobiological methods to predict and intervene in early reading difficulties, thereby advancing scientific progress and theoretical innovation in this field.

Fifthly, the study provides guidance for future research. The findings highlight major trends and unresolved issues in the current research, thereby offering comprehensive guidance and an agenda for future early childhood reading research. These insights are crucial for directing future studies' focus.

#### 5.1.2 Practical implications

At the practical level, this study provides valuable references for practitioners in the field of early childhood reading. Firstly, the findings emphasize the importance of implementing personalized and culturally sensitive educational strategies in practice. In particular, children with special reading needs, such as those with hearing impairments or those from diverse cultural backgrounds, would benefit from educational plans tailored to their specific needs. This study highlights the need for educators to consider individual differences and cultural backgrounds when designing curricula and teaching strategies.

Second, the study shines the spotlight on policy support for diverse educational environments. The research indicates that policymakers need to focus on and support the creation of diverse and inclusive early reading environments by investing in public libraries, community centers, and home reading projects and strengthening these environments through educational policy. Such policy support is essential to ensure that all children have access to high-quality early reading resources.

Thirdly, this study promotes interdisciplinary research. The study's results emphasize the importance of interdisciplinary research in the development of early reading. For instance, the findings have revealed the crucial roles of mathematical abilities, writing skills, and visual and neural activation in early reading development, urging researchers to adopt an interdisciplinary approach that combines knowledge and techniques from linguistics, psychology, neuroscience, and other fields to fully understand reading development.

### 5.2 Future research directions

Based on an in-depth analysis of 11 key topics in the field of early reading, we propose the following future research directions.

One potential future research direction is the digital age and the reading experience. Under Topic 1, we discovered that in the digital age, electronic books, animated books, applications, and online reading platforms can provide children with a brand new reading experience (Pettersen et al., 2022). Research revolving around books is an emerging hot topic in the field of early reading. Although the academic community has already proven that digital technology can cultivate early reading skills in children, it is currently unclear how various factors influence the effectiveness of digital reading intervention measures, such as the type of technology, training content, and integration level. Therefore, further research is needed to explore how early reading interventions' effectiveness is modulated by the latest digital tools, such as augmented reality and virtual reality, as well as game features (Vanbecelaere et al., 2023).

Another potential future research direction is cross-cultural and multilingual reading. With the deepening of globalization, the significance of cross-cultural and multilingual reading has become increasingly prominent. In this context, China, as an emerging global power, has witnessed its culture and language becoming increasingly vital among international scholars. As illustrated regarding Topic 2, recent years have seen a growing focus on the study of Chinese reading and the early reading habits of Chinese children in the academic realm. This attention not only signifies curiosity toward Chinese children's early reading developmental characteristics but also highlights the heightened international recognition of Chinese as a significant language. On the other hand, Topic 5 predominantly explores reading capabilities in a bilingual environment, a topic of pressing importance in today's multilingual and multicultural world. Notably, although research on reading in multilingual contexts, especially those involving English, is abundant, studies on children's reading in a Chinese–English bilingual setting are relatively scarce. Given that both Chinese and English rank among the most influential languages globally, research on their cross-linguistic transfer, bilingual reading development, and cross-cultural reading competencies is undeniably of profound significance to researchers, educators, and globalization itself (Ye et al., 2022a). Therefore, in light of the aforementioned scenarios, future research should place greater emphasis on integrating insights from Topics 2 and 5 to delve deeper into the cross-linguistic reading capabilities and cross-cultural understanding of children in a Chinese–English bilingual context. Such investigations could aim to provide a more in-depth and forward-looking theoretical foundation for bilingual education and cultural exchanges in a globalized setting.

Integrated learning approaches constitute another potential future research direction. In the future, early reading practices will no longer be perceived solely as an isolated activity. As the studies classified under Topics 3 and 4 have illustrated, scholars are increasingly intrigued by the integration and simultaneous development of reading skills and mathematical competencies (Inoue et al., 2023), writing abilities (Otsuka and Murai, 2023), and other associated proficiencies. Such interdisciplinary research approaches aim to amalgamate various learning skills to offer a more holistic and ecological learning system. However, although these domains' convergence presents numerous innovative research opportunities, there is still a notable gap in comprehensive exploration of how such integrated learning methods impact children's holistic development. Thus, the potential effects of an integrated learning approach on children's well-rounded growth will undoubtedly emerge as a pivotal focus in future academic investigations.

Another recommended future research direction is reading performance in special populations. Our research analysis has shown that the core topics, namely Topics 8 and 11, have prominently focused on the reading performance characteristics of two specific child groups: children with early reading disabilities and children diagnosed with early ASD. This research has yielded invaluable data and insights for interventions targeting these special populations' early literacy skills. Presently, the majority of scholars predominantly emphasize the reading characteristics of children with reading disabilities and delve into the prospects of phonological interventions as a means to enhance their reading skills (Lemons et al., 2018). Concurrently, based on observations made regarding Topic 6, there appears to be burgeoning research interest in understanding how external community environments influence reading interventions for children. Although studies in this particular direction are still in their infancy, considering the potential impact of community environments on children with early reading disabilities and early ASD, coupled with the prospective effectiveness of community-based interventions, this research area undoubtedly holds significant promise (Piasta et al., 2023). Consequently, future research endeavors should further investigate how community environments interact with the reading performance characteristics of these unique child groups and develop effective intervention strategies tailored to these characteristics.

Our final recommended future research direction is neuroscience in early reading. Based on the results for Topics 8, 10, and 11, neuroscience methods have evidently been widely applied to study brain activity characteristics during early reading processes. For example, the use of fMRI technology can help us better understand the bidirectional relationship between reading skills and brain speech processing (Wang et al., 2023) and explore the association between early reading skills and the organization of the ventral occipito–temporal cortex (Chyl et al., 2023). The application of neuroscience methods has obviously brought innovation to early reading research. However, we should not overlook the complexity and cost associated with the use of neuroscience techniques. Future research efforts could focus on identifying methods to optimize the cost of using neuroscience technology in the early reading field. Additionally, most scholars engaged in early reading research come from the social science discipline and may be unfamiliar with the procedures and standards for using neuroscience methods. Therefore, it is necessary to conduct a specialized and systematic review of the relevant literature on neuroscience and early reading to fully demonstrate neuroscience technology's application value in early reading.

## 6 Conclusions and limitations

Utilizing the DTM, this study identified and analyzed 11 primary research topics in the field of early reading and revealed a diverse array of subjects and evolving focuses in scholarly inquiry. The research has underscored the profound influence of the digital age on methodologies, tools, and theoretical approaches in early reading research. It has emphasized the critical importance of personalized and culturally sensitive educational strategies in practice and affirmed the necessity of interdisciplinary research for a comprehensive understanding and the promotion of early reading development. Moreover, this study has charted a course for future research by suggesting paths such as exploring reading experiences in the digital age, investigating cross-cultural and multilingual reading practices, and applying neuroscience to early reading. Ultimately, the insights and directives derived from this research have significantly enriched the early reading domain, laying the groundwork for enhanced exploration and deeper investigation into these pivotal topics, thereby promising to shape the future trajectory of early reading research.

However, this study has certain limitations. The research focused solely on literature obtained from the WOS Core Collection, without investigating non-Core Collection literature and that from other databases such as Scopus. Future research could expand the time span and data sources to comprehensively understand the knowledge structure and developmental trends in the field of early reading.

## Data availability statement

The original contributions presented in the study are included in the article/supplementary material, further inquiries can be directed to the corresponding authors.

## Author contributions

TW: Writing – review & editing, Visualization, Methodology, Conceptualization. HX: Writing – original draft, Formal analysis, Methodology, Writing – review & editing. CL: Data curation, Visualization, Writing – original draft. FZ: Data curation, Visualization, Writing – original draft. JW: Formal analysis, Supervision, Writing – review & editing.
